# Systematic evaluation and optimization of the experimental steps in RNA G-quadruplex structure sequencing

**DOI:** 10.1038/s41598-019-44541-4

**Published:** 2019-05-30

**Authors:** Pui Yan Yeung, Jieyu Zhao, Eugene Yui-Ching Chow, Xi Mou, HuiQi Hong, Leilei Chen, Ting-Fung Chan, Chun Kit Kwok

**Affiliations:** 1 Department of Chemistry, City University of Hong Kong, Kowloon Tong, Hong Kong SAR China; 2 School of Life Sciences, and State Key Laboratory of Agrobiotechnology, The Chinese University of Hong Kong, Shatin, Hong Kong SAR China; 3 Department of Physiology, Yong Loo Lin School of Medicine, National University of Singapore, Singapore, 117549 Singapore; 4 Cancer Science Institute of Singapore, National University of Singapore, Singapore, 117599 Singapore; 5 Department of Anatomy, Yong Loo Lin School of Medicine, National University of Singapore, Singapore, 117594 Singapore

**Keywords:** Biological techniques, Nucleic acids

## Abstract

cDNA library preparation is important for many high-throughput sequencing applications, such as RNA G-quadruplex structure sequencing (rG4-seq). A systematic evaluation of the procedures of the experimental pipeline, however, is lacking. Herein, we perform a comprehensive assessment of the 5 key experimental steps involved in the cDNA library preparation of rG4-seq, and identify better reaction conditions and/or enzymes to carry out each of these key steps. Notably, we apply the improved methods to fragmented cellular RNA, and show reduced RNA input requirement, lower transcript abundance variations between biological replicates, as well as lower transcript coverage bias when compared to prior arts. In addition, the time to perform these steps is substantially reduced to hours. Our method and results can be directly applied in protocols that require cDNA library preparation, and provide insights to the further development of simple and efficient cDNA library preparation for different biological applications.

## Introduction

Multitudes of cDNA library preparation methods have been developed as a result of the recent exploration and investigation of the novel features of RNA, such as RNA structures^1–4^. However, most of these methods require high RNA input (microgram of RNA), numerous processing and purification steps, and thus long cDNA library preparation time (few days)^5–9^. As such, a simple and efficient cDNA library preparation is critical to the further improvement of existing protocols, development of new transcriptome-wide methods and many other biochemical assays.

**Figure 1:**
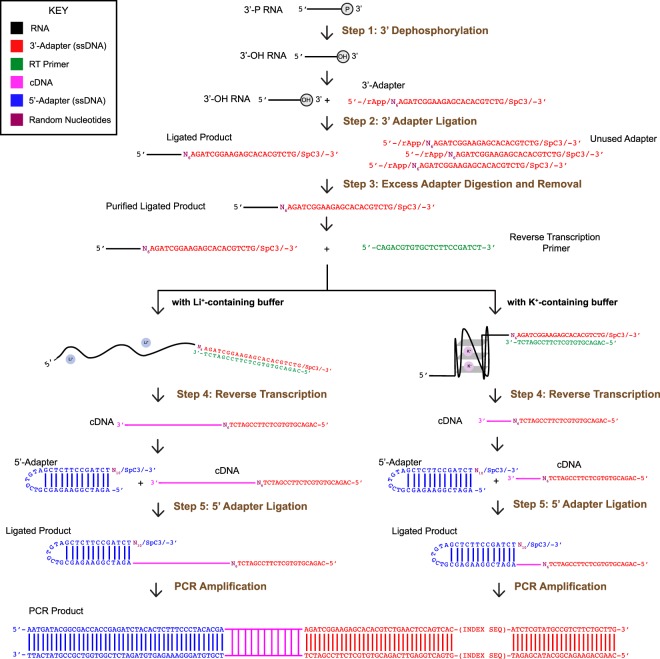
Experimental flowchart of RNA G-quadruplex structure sequencing (rG4-seq). The key experimental steps that have been evaluated and optimized in this work are labeled (steps 1–5). The sequence information of the oligonucleotides used can be found in Table S1. The RNA of interest with a 3′ phosphate can be from synthetic RNA oligos or randomly fragmented RNAs. Dephosphorylation reaction replaces the 3′-P (2′,3′-cyclic-P, 2′P, 3′P ends) with 3′-OH in the RNA (step 1). 3′-adapter is then ligated to the 3′ end of the RNA fragment (step 2). The random nucleotides introduced in the 3′-adapter can reduce ligation bias, and be used as unique molecular index to remove PCR duplicates in the sequencing analysis. Excess 3′-adapter digestion and removal step (step 3) is then performed, so as to remove the unused 3′-adapter during the 3′-adapter ligation step. Reverse transcription (RT) is performed under either Li^+^- or K^+^-containing buffer, of which the latter will cause reverse transcriptase to stall at rG4 region (step 4). After the RT, 5′-adapter (with respect to the final PCR product) is then ligated to the 3′ end of the cDNA (step 5). The random nucleotides introduced in the 5′-adapters can reduce ligation bias. PCR amplification will be performed afterwards to produce double-stranded DNA with 6-bp index sequence involved.

Recently, we have developed RNA G-quadruplex structure sequencing (rG4-seq), and reported the prevalent *in vitro* formation of RNA G-quadruplex structures in the human transcriptome^9^, providing a useful resource for further *in vivo* rG4 structural and functional characterization. The rG4-seq protocol requires high input of RNA (~500 ng RNA), and lengthy library preparation (~1.5 days)^9,10^. In addition, an extensive assessment on the procedures of the experimental pipeline of rG4-seq is currently lacking. In this study, we have systematically evaluated and optimized the 5 key experimental steps of the experimental pipeline in rG4-seq, namely 3′ dephosphorylation, 3′-adapter ligation, excess 3′-adapter digestion and removal, reverse transcription, and 5′-adapter ligation (Fig. 1). We applied the improved methods to fragmented cellular RNA and generated two new cDNA libraries using reduced RNA inputs (~250 ng and ~50 ng RNA). The new libraries were found to have lower transcript abundance variations and lower 5′ and 3′ transcript coverage bias when compared to prior arts^9^. Moreover, the total time of the library preparation has been greatly reduced to several hours.

## Methods

### 3′ dephosphorylation

#### Preparation of N_39_rN for dephosphorylation reaction

Six microliters of 50 μM FAM-labelled N_39_rNN_3_ chimera oligo of DNA and RNA was first mixed with 44 μl of nuclease-free water and treated with 12.5 μl of 2 M sodium hydroxide (NaOH) at 95 °C for 10 minutes. This step cleaved the N_39_rNN_3_ chimera oligo at the RNA (rN) position to generate N_39_rN that mimics the 3′ end functional groups after fragmentation in the real library preparation. After the RNA degradation treatment, column purification was carried out to remove the NaOH and obtain the purified N_39_rN chimera oligo by eluting with 10 μl nuclease-free water.

#### Dephosphorylation reaction

T4 PNK (NEB, M0201S), Fast AP (Thermo Scientific, EF0654), and rSAP (NEB, M0371S) were used to compare their efficiency on the dephosphorylation of purified N_40_ chimera oligo. Since the reaction rate was fast in the original enzyme concentration, the three enzymes were diluted 10-fold dilution for the reaction. The reaction was 10 μl in total including 8 μl of FAM-labelled cleaved N_40_ chimera oligo (0.1 μM final), 1 μl of 10X T4 PNK reaction buffer (1U/μl), 1 μl of 10-fold diluted T4 PNK (0.1U/μl final), Fast AP (0.01U/μl final) or rSAP (0.01U/μl final) and was performed at 37 °C for 0, 1, 5, 15 and 30 minutes.

To determine the dephosphorylation efficiency, 8% denaturing gel electrophoresis containing 7.5 M of urea was conducted. The reaction was first quenched by adding 1 volume of 2X formamide orange G dye (94% deionized formamide, 20 mM Tris pH 7.5, 20 mM EDTA, orange G dye). After pre-heating the gel (90 W for 45 minutes), samples from each reaction were inactivated at 95 °C for 3 minutes before loading 3.2 μl to each well for electrophoresis at constant 90 W for 90 minutes. As the N_40_−3′P/2′P/2′3′cyclic P could run faster than N_40_-3′OH due to the presence of negative change, the % yield could be quantified directly^11^. The gel was directly scanned by Fujifilm FLA 9000 Imager (fluorescence detection mode), and analysed (see Data processing and analysis).

### 3′-adapter ligation reaction

The reaction mixture consisted of 1 μl of 1 μM N_40_-3′OH RNA (0.1 μM final), 1 μl of 1, 2.5, 5 or 10 μM of 3′-adapter (0.1, 0.25, 0.5 or 1 μM final), 0 or 3.5 μl of 50% polyethylene glycol (PEG) 8000 (0% or 17.5% final), 1 μl of 10X reaction buffer that gave final working concentration of 50 mM Tris-HCl (pH 7.5), 10 mM MgCl_2_, 1 mM dithiothreitol (DTT), and 200 U T4 RNA ligase 2, truncated KQ ligase (NEB, M0373S). This reaction was 10 μl in total and performed at 25 °C for 60 minutes.

To investigate the effect of PEG concentration on the ligation efficiency, 1 μl of 1 μM N_40_-3′OH RNA (0.1 μM final) was reacted with 1 μl of 10 μM of 3′-adapter (1 μM final), under different percentage of PEG 8000. Final PEG concentrations (0, 2.5, 7.5, 12.5, 17.5, 22.5 or 27.5%) were created by adding 0, 0.5, 1.5, 2.5, 3.5, 4.5 or 5.5 μl of PEG 8000 into the reaction respectively. One microliter of 10X reaction buffer (stated as above) and 1 μl of 200U/μl T4 RNA ligase 2, truncated KQ ligase was added. Finally, the reaction mixture was made up to 10 μl with nuclease-free water. The reaction conditions were the same as that stated as above.

The effect of PEG types on the ligation efficiency was also being investigated. The 17.5% PEG concentration was selected due to its high efficiency showed in the results from below. The reaction mixture was then prepared, and performed accordingly based on the stated conditions, except that PEG 4000, PEG 6000 and PEG 8000 were used individually.

### Excess 3′-adapter digestion and removal

Ten microliters of the reaction mixture from 3′-adapter ligation was then used for enzymatic digestion and removal of the excess 3′-adapter. Enzymatic digestion was conducted by adding 1 μl of 50 U/μl of 5′ deadenylase (50 U final) (NEB, M0331S) and 1 μl of 30 U/μl RecJf (30U final) (NEB, M0264S) directly into the sample. The enzymatic reaction was performed at 30 °C for 60 or 90 minutes. To carry out both enzymatic digestion and column purification steps, after the incubation, 28 μl of nuclease-free water was added to the reaction mixture to dilute the PEG concentration, and then purified with RNA Clean & Concentrator™ (Zymo Research) by following manufacturer’s protocol. The final elution volume was 10 μl. For the column purification only treatments, the samples were directly subjected to RNA Clean & Concentrator™ after the addition of 30 μl of nuclease-free water to dilute the PEG concentration, ceteris paribus.

### 5′-adapter ligation reaction

The Quick Ligation™ Kit purchased from NEB (M2200S) was used in the reaction, which consisted of 1 μl of N_60_ ssDNA (0.1 μM final), 1 μl of 1, 2.5, 5 or 10 μM of 5′-adapter (0.1, 0.25, 0.5 or 1 μM final), 1 μl of 10X DNA ligase buffer with final working concentration of 66 mM Tris-HCl (pH 7.6), 10 mM MgCl_2_, 1 mM DTT, and 1 mM ATP. After a short vortex and spin down, the reaction mixture with 9 μl in total was then heated at 95 °C for 3 minutes, 60 °C for 1 minute and 25 °C for 2 minutes, then cooled down to room temperature before adding 1 μl of 2000 U/μl of T4 DNA ligase (2,000 U final), and performed at 37 °C (optimal temperature for T4 DNA ligase^12^) for 2 hours.

To investigate the effect of PEG concentration on the ligation efficiency, 1 μl of 1 μM N_60_ ssDNA (0.1 μM final) was reacted with 1 μl of 10 μM 5′-adapter (1 μM final), under different percentage of PEG 6000. Final PEG concentrations (0, 2.5, 7.5, 12.5, 17.5, 22.5 or 27.5%) were created by adding 0, 0.5, 1.5, 2.5, 3.5, 4.5 or 5.5 μl of PEG 6000. Instead of 2X reaction buffer, 10X reaction buffer was made based on the original component to maintain the mixture volume within 10 μl. Then, 1 μl of 10X reaction buffer (stated as above) was added and the reaction mixture was made up to 9 μl with nuclease-free water. 1 μl of 2000 U/μl of T4 DNA ligase was then added. The reaction conditions were the same as that stated as above.

The effect of PEG types on the ligation efficiency was also being investigated. The 17.5% PEG concentration was selected due to its high efficiency showed in the results from below. The reaction mixture was then prepared, and performed accordingly based on the stated conditions.

All reactions listed above were quenched by adding 1 volume of 2X formamide orange G dye (94% deionized formamide, 20 mM Tris pH 7.5, 20 mM EDTA, orange G dye). Samples from each reaction were heated at 95 °C for 3 minutes before loading 2 μl to 10% polyacrylamide-urea denaturing gel containing 8.3 M of urea. The pre-heated gel (300 V for 20 minutes) was subjected to electrophoresis at constant 300 V for 20 minutes. Next, 5 μl of SYBR Gold (Thermo, S11494) was mixed with 50 mL nuclease-free water. The gel was stained by SYBR Gold for 5 minutes, and then directly scanned by Bio-rad ChemiDoc™ Touch Imaging System and the gel image was analyzed (see Data processing and analysis).

### Reverse transcription

The reaction mixture consisted of 1 μl of 1 μM pre-miRNA 149 wild-type (0.1 μM final), 1 μl of 1 μM reverse transcription primer with Cy5 fluorescent tag at 5′ end (0.1 μM final), 3 μl of 5X Li^+^ or K^+^ reverse transcription buffer, which gave a final concentration of 20 mM Tris (pH 7.5), 4 mM MgCl_2_, 1 mM DTT, 0.5 mM dNTPs, 150 mM LiCl or 150 mM KCl or vendor supplied/recommended reaction buffer (SuperScript III: Thermo Scientific, 18080093: 50 mM Tris-HCl (pH 8.3), 75 mM KCl, 3 mM MgCl_2_ and 0.5 mM dNTPs final; ProtoScript II: NEB, B0368S: 50 mM Tris-HCl (pH 8.3), 75 mM KCl, 3 mM MgCl_2_ and 0.5 mM dNTPs final; TGIRT: 20 mM Tris-HCl (pH 7.5), 5 mM MgCl_2_, 450 mM NaCl and 0.5 mM dNTPs final), 4.5 μl of nuclease-free water, and 0.5 μl of 200U/μl SuperScript™ III Reverse Transcriptase (100U final) (Thermo Scientific, 18080093), 200U/μl TGIRT™-III Enzyme (100U final) (InGex), or 200U/μl ProtoScript® II Reverse Transcriptase (100U final) (NEB, M0368S). The guanosine (G) sequencing reaction was prepared by addition of 1 μl of 10 mM dideoxycytidine (ddC) (final 1 mM ddC) instead of nuclease-free water, ceteris paribus. The total volume of the reaction mixture was 10 μl.

The reaction mixture was incubated in a thermal cycler for reverse transcription. Denaturation was performed at 75 °C for 3 minutes, followed by annealing at 35 °C for 5 minutes. The reverse transcriptase was then added and the cDNA synthesis was carried out at 50 °C for 15 minutes. Next, 0.5 μl of 2 M NaOH was added after the reaction to promote hydrolytic degradation of the RNA templates and the reaction was terminated at 95 °C for 10 minutes.

The reaction was quenched by adding 1 volume of 2X formamide orange G dye (94% deionized formamide, 20 mM Tris pH 7.5, 20 mM EDTA, orange G dye) and was heated at 95 °C for 3 minutes. Two microliters of sample were loaded into 8% denaturing polyacrylamide gel containing 8.3 M of urea. The pre-heated gel was subjected to electrophoresis at constant 90 W for 90 minutes. The gel was directly scanned by Fujifilm FLA 9000 Imager (fluorescence detection mode), and the gel image was analysed (see Data processing and analysis).

### Data processing and analysis

#### 3′ dephosphorylation

The background-corrected N_39_rN oligo with phosphate group (P) and the dephosphorylated oligo (OH) were quantified by ImageJ. The percentage of yield was calculated using the equation listed below.

1 $$Percentage\,yield=\frac{P}{P+OH}\times 100 \% $$

#### Ligation reactions

The background-corrected unligated RNA template bands (U) and ligated product (L) bands were quantified by Image Lab™ 6.0.1. The percentage of yield was calculated using the equation listed below.

2 $$Percentage\,yield=\frac{L}{U+L}\times 100 \% $$

#### Excess adapter digestion and removal

The background-corrected ligated product (P) and adapter (A) bands were quantified by Image Lab™ 6.0.1. The reduction percentage was calculated using the following equation. The bands from negative control (untreated sample) and treatment group were annotated as U and T respectively as follows.

3 $$Reduction\,percentage=[1-({P}_{U}/{A}_{U})/({P}_{T}/{A}_{T})]\times 100 \% $$

### Total RNA extraction and polyA RNA enrichment

Authenticated HeLa cells with no mycoplasma contamination were cultured in DMEM (Thermo Scientific, 10569044) media supplemented with 10% heat inactivated fetal bovine serum (Thermo Scientific, 10270106) and 1X antibiotic antimycotic (Thermo Scientific, 15240062) at 37 °C in a humidified incubator with 5% CO_2_.

Cells were harvested for total RNA extraction using RNeasy Plus Mini Kit (Qiagen, 74136) following the manufacturer’s protocol. The concentration of the extracted total RNA was quantified by Nanodrop ND-1000 Spectrophotometer.

PolyA RNAs were purified from 100 µg and 20 µg of total RNA in each biological replicate using the Poly(A)Purist™ MAG Kit (Thermo Scientific, AM1922) according to the manufacturer’s instructions. After 2 rounds of polyA selection, four biological replicates of 500 ng and 100 ng of polyA enriched RNA were used for random RNA fragmentation step (see below).

### Random RNA fragmentation

In each fragmentation reaction, 40 µl of purified RNA (500 ng and 100 ng) were mixed with 10 µl of 5X alkaline fragmentation buffer with a final 1X concentration including 40 mM TrisHCl, pH 8.2, 100 mM LiCl, 30 mM MgCl_2_ and incubated at 95 °C for 60 seconds, and then purified with RNA Clean & Concentrator™ (Zymo Research) by following manufacturer’s protocol. Fragmented RNAs were quantified and characterized by using High Sensitive RNA ScreenTape on the 4200 Tapestation (Agilent Technologies) according to the manufacturer’s protocol.

### Library preparation

Subsequent steps including 3′ dephosphorylation, 3′-adapter ligation reaction, excess 3′-adapter digestion and removal, reverse transcription and 5′-adapter ligation reaction with a detailed method description were showed above. Please note that the reactions should be conducted in the order showed in Fig. 1. RNA samples were split into half for reverse transcription under Li^+^ and K^+^ conditions, the final RNA input for each sample was 250 ng and 50 ng polyA enriched RNA respectively. Before ligating to the 5′-adapter, cDNA samples were purified with RNA Clean & Concentrator™ (Zymo Research) by following manufacturer’s protocol.

After 2 hours of 5′-adapter ligation reaction, the cDNA products were mixed with 2X formamide orange G dye (94% deionized formamide, 20 mM (Tris pH 7.5), 20 mM EDTA, orange G dye) and briefly denatured, then loaded into a 12-well 10% denaturing urea-TBE acrylamide gel (Thermo Scientific, EC68752BOX) and run at 300 V for 20 minutes with the low molecular weight DNA ladder (NEB, N3233S). The gel was stained with SYBR Gold (Thermo, S11494) for 5 minutes, then the 100–400 nt region in each sample was excised. The gel was physically disrupted and followed by adding 320 µl of 1X TEN250 buffer (1X TE 7.4, 0.25 M NaCl) with incubation at 80 °C with 1300 rpm shaking for 30 minutes. The gel debris was removed from the eluate using a Spin-X column (Corning, 8160) and then purified with RNA Clean & Concentrator™ (Zymo Research) by following manufacturer’s protocol.

### PCR amplification and analysis

The reaction mixture consisted of 8 μl of sample cDNA from 5′-adapter reaction, 1 μl of 10 μM forward primer (Table S1), 1 μl of 10 μM reverse primer (Table S1) and 10 μl of 2X KAPA HiFi HotStart ReadyMix (KAPA Biosystems, KK2602). The reaction mixture was incubated in thermal cycler for PCR amplification. Initial denaturation was performed at 95 °C for 3 minutes, followed by the amplification step with desired number of cycles (12 cycles for 250 ng input and 19 cycles for 50 ng input). The amplification cycling program comprises denaturation at 98 °C for 20 seconds, annealing at 68 °C for 15 seconds, extension at 72 °C for 40 seconds. The samples were heated at 72 °C for 90 seconds, and finally cooled down to 4 °C for size-selection.

PCR samples were added with 6X orange loading dye (NEB, B7022S) and then resolved on a 1.8% TAE agarose gel at 120 V, 55 min with 4 μl Quick-load Purple LMW marker (NEB, N0557S) included. The 150–400 nt region of each PCR product was cut and purified by using the Zymo DNA agarose gel extraction kit (Zymo, D4008) following manufacturer’s protocol.

To determine the concentration and length of the PCR products obtained, the Agilent D1000 tape operation was performed on TapeStation 4200. One microliter of the PCR product was aliquoted and mixed with 3 μl D1000 sample buffer. After being vortexed and spun down, the reaction mixture was set into the 4200 Tapestation (Agilent Technologies) and the program was operated according to the manufacturer’s instruction.

Ten microliters of qPCR reaction was performed on the 10000X dilutions of each library in water with KAPA Universal Quant Kit (KAPA, KK4824) following the manufacturer’s instructions for library quantification before subjecting to NGS. Quantified DNA libraries were pooled and then sequenced on the Illumina Hiseq System in 150 bp paired-end (PE) configuration.

### Sequencing data processing and analysis

Sequencing data were preprocessed by Cutadapt (version 2.1)^13^ for removal of illumina sequencing adapters and low-quality bases. Trimmed data were aligned to human reference genome version hg19 using STAR (version 2.7.0)^14^. Human reference genome and gene annotation files were downloaded from Illumina iGenomes support website (https://support.illumina.com/sequencing/sequencing_software/igenome.html). Unique molecular identifier (UMI) barcode extraction and subsequent reads deduplication (using uniquely-aligned reads only) were conducted using UMI-tools (version 1.0.0)^15^. Transcript coverage bias was evaluated using Qualimap (version 2.2.1)^16^. Transcript abundances were quantitated as transcript per million (TPM) using Kallisto (version 0.43.1)^17^. Transcripts of log_2_(TPM + 1) ≥ 2 were retained for hierarchical clustering analysis using Python. For new rG4-seq libraries (HeLa-new-250ng, HeLa-new-50ng), only first-in-pair reads were used for coverage bias and transcript abundance analysis.

## Results and Discussion

### Optimization of the RNA 3′ dephosphorylation step

**Figure 2:**
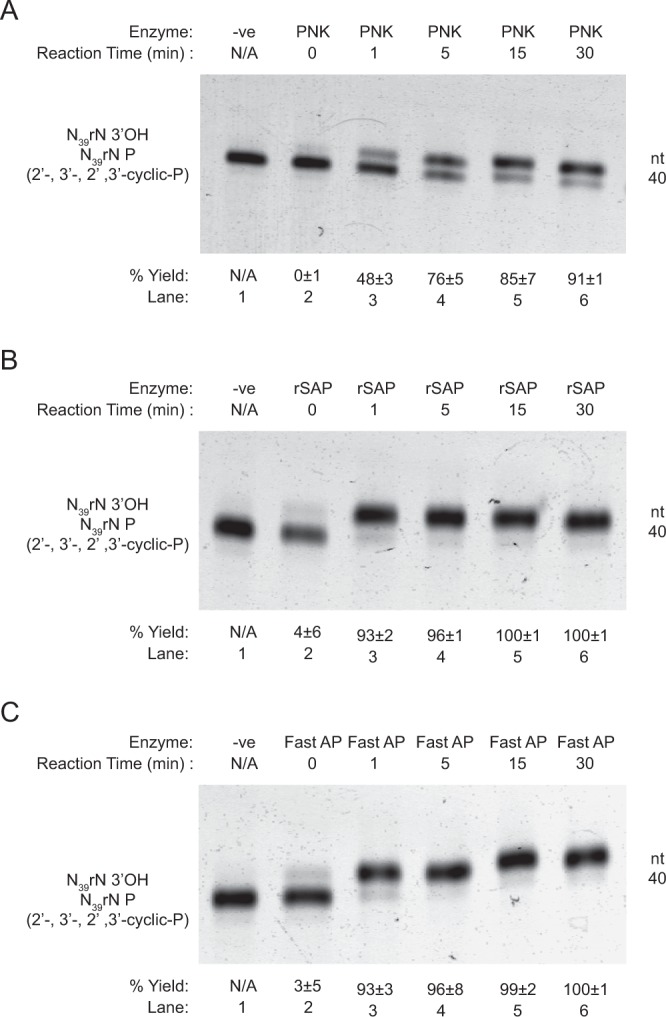
The efficiency of different enzymes on dephosphorylation. 40-nt N_39_rN 3′-P oligonucleotide (with 5′ OH and 2′,3′-cyclic-P,2′P, 3′P ends) was performed by three enzymes, (**A**) T4 PNK, (**B**) rSAP and (**C**) Fast AP. The reactions were performed at 5 different time-points. The dephosphorylation efficiency was enhanced through longer reaction time (panel A-C, lanes 1–6). Furthermore, as the data suggested, PNK showed a slower efficiency than the other two enzymes. Equation 1 was used for the % yield calculation (See Methods). Errors shown were standard deviation. nt = nucleotide; n = 3. The full-length gels are presented in Supplementary Fig. 1.

To evaluate the efficiency of the 3′ dephosphorylation step (Fig. 1, step 1), and to mimic the end-products of the RNA sample being randomly fragmented in the rG4-seq library preparation^9^, we prepared a 40-nt randomized oligonucleotide, N_39_rN-3′P, which contains 2′,3′cyclic-P, 3′P and 2′P after RNA fragmentation (See Methods and Table S1). In the original rG4-seq work^9^, T4 PNK was used as it has been previously reported to have 3′ dephosphorylation activity in the absence of ATP^11^, and has been used in several RNA structurome studies^18–20^. Besides T4 PNK, two other enzymes, rSAP and Fast AP, have also been used for the 3′ dephosphorylation step in RNA structurome studies^8,21^; however, to our knowledge, no study has ever reported and compared their 3′ dephosphorylation efficiency. To examine and improve this 3′ dephosphorylation step for rG4-seq and RNA structurome studies, we tested and compared the three different enzymes, T4 PNK, rSAP, and Fast AP on model oligonucleotide N_39_rN-3′P (Fig. 2).

We performed the dephosphorylation at different time points (0, 1, 5, 15, 30 minutes), and ran them on long denaturing PAGE gel to separate the 3′P reactant and 3′OH product. Compared to rSAP and Fast AP, T4 PNK showed slower dephosphorylation efficiency in general (Fig. 2). For example, rSAP reached to completion (i.e. all reactant has been converted to product) at 15 minutes (Fig. 2B, lane 5), whereas that of Fast AP happened at around 15 to 30 minutes time (Fig. 2C, lane 5 and 6). However, the maximum production yield of PNK was 91 ± 1 at 30-minute timepoint (Fig. 2A, lane 6). Therefore, we can conclude that the dephosphorylation efficiency of T4 PNK was relatively lower than the other two enzymes.

Overall, we have identified rSAP and FastAP to be more efficient than T4 PNK in 3′ dephosphorylation, and showed that the reaction was nearly quantitative within 30 minutes at 37 °C (Supplementary Fig. 1). Since these enzymes work under same reaction buffer, it is possible to use them in combination.

### Optimization of the 3′-adapter ligation step

To examine the efficiency of 3′-adapter ligation (Fig. 1, step 2), we have used synthetic RNA oligonucleotide, N_40_-3′OH (Table S1), and assessed three factors, including ratio of RNA to adapter, PEG 8000 concentration, and PEG type. To monitor the ligation efficiency, the N_40_-3′OH was ligated with the 28-nt 3′-adapter (Table S1) using T4 RNA ligase 2, truncated KQ, which has been demonstrated to perform better than T4 RNA ligase 1^22^. Subsequently, denaturing PAGE was performed to size fractionate the ligated and unligated products.

**Figure 3:**
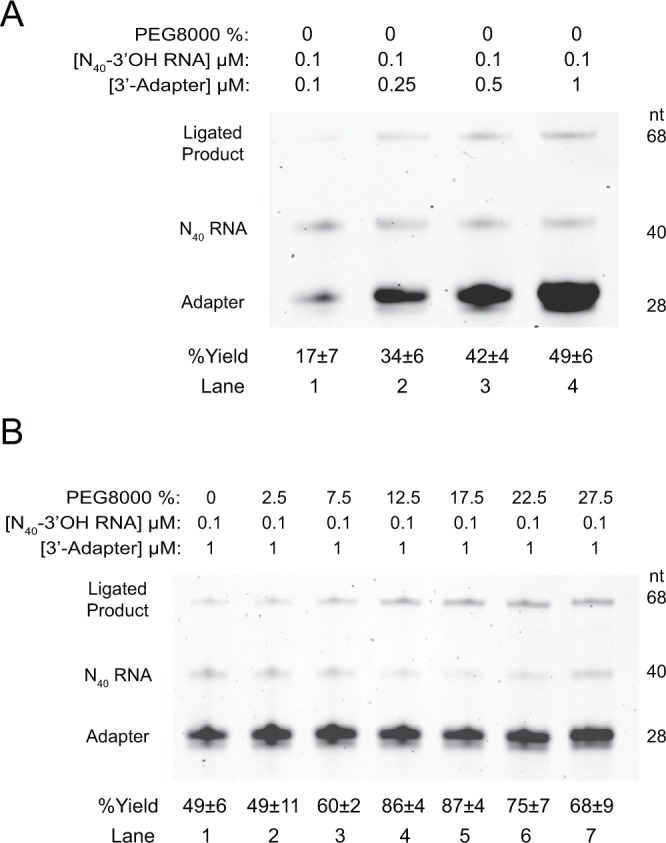
The effect of 3′-adapter concentration and different percentages of PEG 8000 on the 3′-adapter ligation efficiency. The figure showed the efficiency of 3′-adapter ligation performed by T4 RNA Ligase 2, truncated KQ with varying (**A**) RNA:adapter ratio, from 1:1 to 1:10, or (**B**) the percentages of PEG 8000, from 0% to 27.5%. The 40-nt RNA N_40_-3′-OH (with 5′OH and 3′OH ends) was ligated with the 28-nt 3′-adapter (with 5′rApp and 3′C3 spacer ends) to produce the 68-nt ligation product. The ligation yield enhanced with increasing 3′-adapter concentration (panel A, lane 1–4). From 0% to 17.5% PEG 8000 concentration, the ligation yield increased with increasing PEG 8000 concentration (panel B, lanes 1–5), but started to drop afterwards (panel B, lanes 6 and 7). The ligation yield was the highest at 12.5–17.5% PEG 8000 condition in 1:10 RNA:adapter ratio. Equation 2 was used for the % yield calculation (See Methods). Errors shown were standard deviation. nt = nucleotide; n ≥ 3, The full-length gels are presented in Supplementary Fig. 2.

First, we tested the effect of RNA to adapter ratio on 3′-adapter ligation, as we anticipated that the relative concentrations of the two would influence ligation efficiency. Four RNA:adapter ratios ranging from 1:1 to 1:10 were performed using the same condition (see Methods). Generally, the production yield increased with decreasing RNA:adapter ratio (Fig. 3 and Supplementary Fig. 2). In other words, the more 3′-adapter added in the reaction mixture, the more efficient the RNA ligation reaction was. For the 0% PEG 8000 condition, 1:1 ratio yielded 17 ± 7% ligation efficiency (Fig. 3A, lane 1), whereas the 1:2.5, 1:5, and 1:10 ratio yielded 34 ± 6%, 42 ± 4%, and 49 ± 6% respectively (Fig. 3A, lanes 2–4).

Second, we reasoned that PEG 8000 concentration might affect the ligation efficiency, as previous study has reported it to enhance the performance of T4 RNA ligase^23^. To test this, we have performed the 3′-adapter ligation reaction under different concentrations of PEG 8000, ranged from 0 to 27.5%, in 1:10 of RNA:adapter ratio. Our results showed that the ligation yield with PEG 8000 addition (Fig. 3B) were generally higher than the one with no PEG 8000 addition (Fig. 3A), and was the highest with 12.5% (86 ± 4%) to 17.5% (87 ± 4%) PEG 8000 (Fig. 3B, lanes 4–5). Consistent with the case for Fig. 3A, we observed increased ligation yield with decreasing RNA:adapter ratio in the presence of 17.5% PEG 8000 (Supplementary Fig. 3).

Last, the effect of PEG types on the 3′-adapter ligation efficiency was investigated (Supplementary Fig. 4). Three types of PEG with different average molecular weight, PEG 4000, 6000 and 8000 were used under 1:10 RNA:adapter ratio. The data suggested the 3′-adapter ligation yields were all above 80%, however, no significant difference was observed between the three PEG types (Supplementary Fig. 4).

In sum, we found that T4 RNA ligase 2, truncated KQ was efficient in the 3′-adapter ligation with 12.5% to 17.5% of PEG 8000 and 1:10 RNA:adapter ratio, with production yield more than 80% within 1 hour at 25 °C.

### Optimization of the excess 3′adapter digestion and removal step

To remove the excess 3′-adapter after the 3′-adapter ligation step (Fig. 1, step 2), 3′-adapter digestion and/or clean up procedures were performed to remove the excess adapter (Fig. 1, step 3), in order to reduce the carry-over contamination that might affect downstream steps such as reverse transcription (Fig. 1, step 4). Altogether, three approaches have been examined as described below.

**Figure 4:**
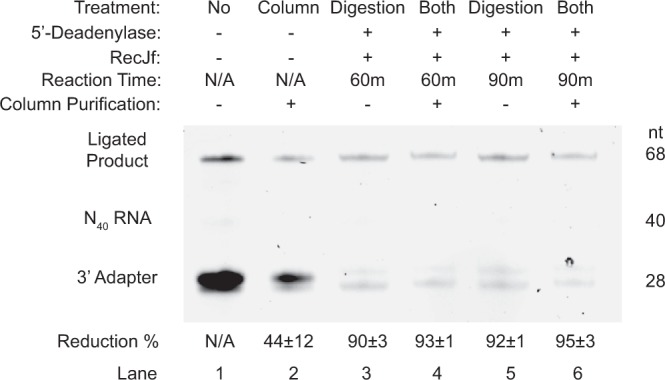
The effect of enzymatic digestion and column purification on excess 3′-adapters. The efficiency of the three approaches to remove the excess 3′-adapter (lane 1), column purification only (lane 2), enzymatic digestion for 60 minutes and 90 minutes (lanes 3 and 5), and column purification after enzymatic digestion (lane 4 and 6), were shown. After ligation of N_40_ RNA (40 nt) with 3′-adapter (28 nt) to form the ligated product (68 nt), unused adapters remain and need to be removed for subsequent reactions. As compared with no treatment sample in lane 1, column purification alone was able to remove over 40% of excess adapters, while adopting the digestion (lanes 3 and 5) or both digestion and column purification (lanes 4 and 6) removed more than 90% of excess adapters. Furthermore, if the reaction time increased from 60 minutes to 90 minutes, the reduction % enhanced slightly (lanes 3 and 5). Equation 3 was used for reduction % calculation (see Methods). Note that the reduction was calculated by ratio of ratio (see Methods, Eq. 3), thus the difference observed in the gel image was unaffected by the loading error. The reduction percentage of each treatment was shown with the standard deviations indicated. nt = nucleotide; n = 3. The full-length gel is presented in Supplementary Fig. 3.

For the first approach, we have introduced a new enzymatic digestion step, which is absent in the original rG4-seq protocol. 5′deadenylase was first used to remove the 5′-rApp functional group of the excess 3′-adapter, leaving the adapter with a 5′ monophosphate. Next, specific 5′-3′ DNA exonuclease RecJf was applied to enzymatically digest the unligated adapter, which is made up of DNA. As the ligated adapter is linked to the RNA, it is not affected by the two enzymes added. Also, the RecJf is a DNA-specific exonuclease and will not digest RNA from the 5′-3′ direction as well. This 5′ deadenylase and RecJf enzymatic digestion has been reported previously in ribosome profiling assay^24^, however, its efficiency has not been reported. Our results showed that this approach resulted in 90 ± 3% and 92 ± 1% reduction in 60 minutes and 90 minutes reaction at 30 °C respectively (Fig. 4, compare lane 1 with lanes 3 and 5). Note that the reduction % was calculated by ratio of ratio, which account for loading error (see Methods, Eq. 3). We also noted that 60 minutes of enzymatic treatment has already reached about 90% reduction in excess adapter, however, longer reaction time (e.g. 90 minutes) might be used to further reduction of the residual excess adapter.

For the second approach, we attempted to clean up the excess adapters using column purification with an estimated RNA size cutoff at about 17 nt. Since our 3′-adapter is 28 nt in length and our ligated product is at 68 nt, we expected that the reduction in the 3′-adapter band to be higher than the ligated product band, thus achieving the preferential removal of excess adapters. Our result suggested that this is indeed the case, as this approach resulted in 44 ± 12% reduction (Fig. 4, compare lanes 1 and 2).

For the last approach, we combined both strategies above and performed both enzymatic digestion and column purification, and yielded almost quantitative reduction yield of 93 ± 1% and 95 ± 3% for the samples treated with enzymes for 60 minutes and 90 minutes respectively (Fig. 4, compare lane 1 to lanes 4 and 6). In short, we identified that the removal of excess 3′-adapter was robust when using the enzymatic digestions (>90%) (Supplementary Fig. 5), and can be coupled with column purification to clean up the enzymes and exchange buffer for further downstream processing (Fig. 1, step 4–5)

### Optimization of the reverse transcription step

To optimize the reverse transcription step (Fig. 1, step 4), we used the precursor microRNA 149 (pre-miR149) (Table S1), which has been shown to fold into an RNA G-quadruplex (rG4) under K^+^ and to an RNA hairpin structure under Li^+^ condition^25^ as the RNA template, and a Cy5-fluorescently labeled DNA oligonucleotide as reverse transcription primer (Table S1). Both the buffer conditions and different reverse transcriptases were comparatively tested.

**Figure 5:**
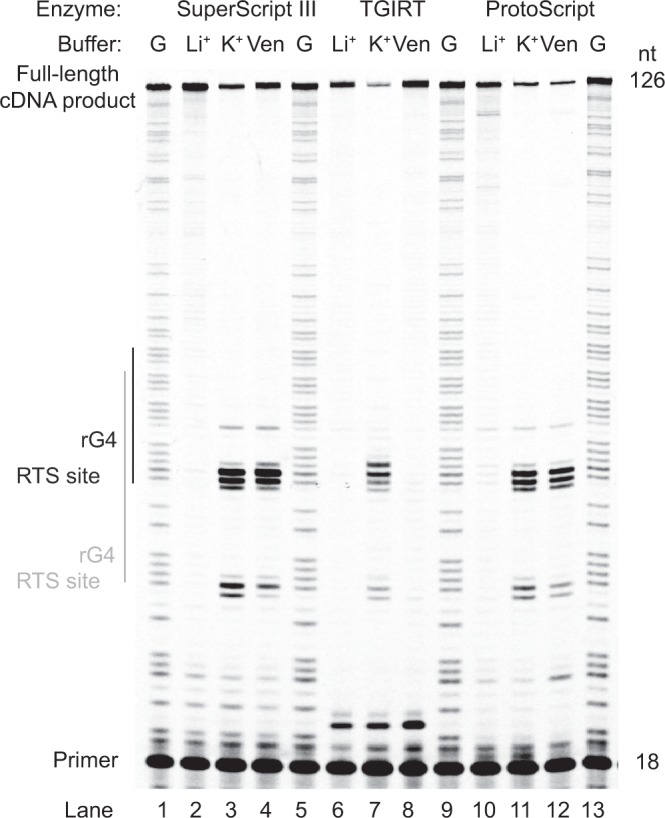
The effect of different monovalent ion-containing buffers and enzymes on the reverse transcription for pre-miRNA 149 sequence. The reverse transcription was conducted with Li^+^-, K^+^-containing and vendor-supplied/recommended buffer from SuperScript III (SSIII) (lanes 2–4), TGIRT (lanes 6–8) and ProtoScript (lanes 10–12). Dideoxycytidine sequencing was performed for guanosine (G) assignment. (lanes 1, 5, 9, 13). The full-length cDNA product band is at 126 nt. The primer is at 18 nt. Reverse transcriptase stalling (RTS) induced by RNA G-quadruplex (rG4) formation in pre-miRNA 149 was observed in both K^+^ and vendor-supplied buffer for SSIII and ProtoScript, yet, only K^+^ buffer contributed to the RTS for TGIRT. The full-length gel is presented in Supplementary Fig. 4.

We initially tested the different reverse transcription buffer conditions that could allow most efficient reverse transcription. To achieve this, we performed reverse transcription under Li^+^ condition, and compared with K^+^ under same ionic strength and also with buffer provided by the SSIII (Fig. 5, lanes 1–4, see Methods). Our results showed that under K^+^ and vendor-supplied buffer (contains K^+^), reverse transcriptase stalling (RTS) was observed in pre-miR149 that is 3′ to the site of rG4 (Fig. 5, lanes 3–4), which is similar to other rG4 examples that we reported previously^9,26^. This suggested that K^+^ and vendor-supplied buffer should be used with great caution for transcripts that contain rG4, depending on the design and application of the assay. Under Li^+^ condition in which rG4 is not stabilized, we observed no RTS, which allowed more full-length product to be produced as compared to under K^+^ and vendor-supplied buffer (Fig. 5, compare lanes 2–4), suggesting that Li^+^ condition is preferred if full-length product is desired. This result also suggested that RNA hairpins that form under Li^+^ condition, as reported previously^25^, do not cause observable RTS (Fig. 5, lane 2).

We further tested and compared the SSIII results with two other commonly used reverse transcriptases, TGIRT and ProtoScript, and investigated their reverse transcription efficiency under Li^+^, K^+^ and vendor-supplied/recommended buffer conditions. Three key findings were observed. First, we found that our Li^+^-containing buffer works well for all 3 different reverse transcriptases, producing mainly full-length bands and no observable stalling at the rG4 site (Fig. 5, lane 2, 6 and 10). Second, under K^+^ condition, all three reverse transcriptases showed RTS at the rG4 site, indicating these reverse transcriptases could recognize rG4 as the roadblock in reverse transcription (Fig. 5, lane 3, 7 and 11). Third, the SSIII and Protoscript reverse transcriptases both caused RTS under vendor-supplied buffer, whereas the TGIRT reverse transcriptase did not cause RTS under its corresponding vendor-recommended buffer (Fig. 5, lane 4, 8 and 12). By inspecting the composition of the vendor-recommended buffer, we found that the SSIII and Protoscript buffers contain K^+^, thus behaving similarly to the K^+^-containing buffer that we used (see Methods). For TGIRT’s buffer, it contains Na^+^, and although Na^+^ was reported to stabilize rG4, albeit less compared to K^+^^27^, the stabilization is not sufficient to cause RTS in this example. Overall, we found that for all the three reverse transcriptases tested, the Li^+^- containing buffer condition is preferred if full-length cDNA is desired, and the K^+^-containing buffer condition is useful in the detection of the rG4 location (Supplementary Fig. 6).

### Optimization of the 5′-adapter ligation step

To assess the efficiency of the 5′-adapter ligation step (Fig. 1, step 5), we have designed a 60-nt randomized DNA oligonucleotides, N_60_, with a 5′ and 3′ hydroxyl group to mimic the cDNA product from reverse transcription step, and ligated to a 44-nt single stranded DNA (ssDNA) adapter containing a 5′ phosphate and 3′ C3 spacer (Table S1) to yield a ligated product of 104-nt in length with the help of T4 DNA ligase. Note that we define this adapter to be 5′-adapter, as this will be at the 5′ side of the final dsDNA PCR product (see Fig. 1). The 5′-adapter has been modified slightly from our previous studies^9,26^ to include a N_10_ templating region for the hybridization-based DNA ligation^28^. Three factors were examined, including the ratio of cDNA to adapter, PEG concentration and the PEG type.

**Figure 6:**
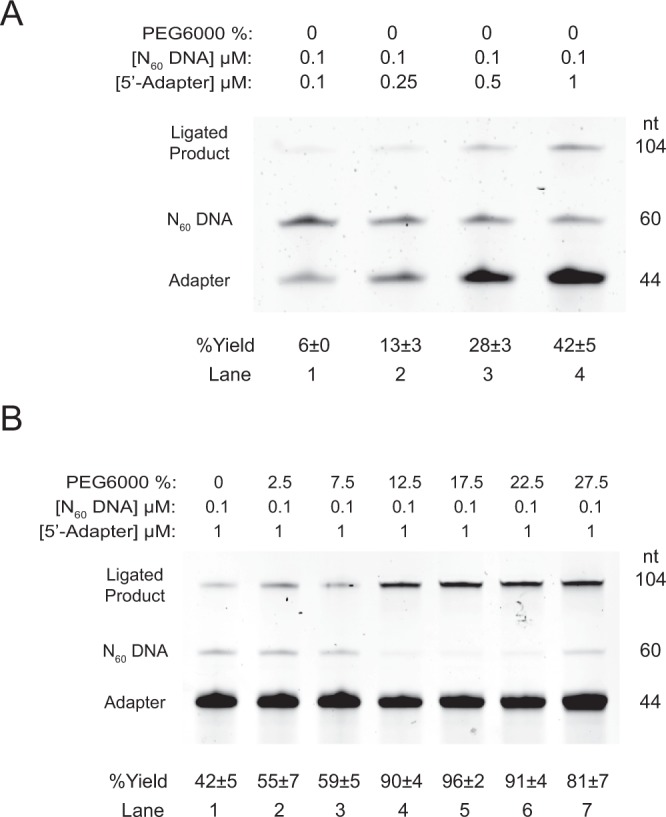
The effect of 5′-adapter concentration and different PEG 6000 concentrations on the 5′-adapter ligation efficiency. The 104-nt ligated products were produced by T4 DNA ligase which either vary in (**A**) cDNA: adapter ratio, from 1:1 to 1:10, or (**B**) PEG 6000 precentages, ranged from 0% to 27.5%. The 60-nt N_60_-DNA (with 5′OH and 3′OH ends) was ligated with 44-nt 5′-adapter (with 5′P and 3′C3 spacer ends) to produce 104-nt ligation products. The ligation yield increased with increasing 5′-adapter concentration (panel A, lanes 1–4). Based on the results from 0% to 17.5%, the ligation efficiency increased with increasing PEG concentration (panel B, lane 1–5), but started to drop afterwards (panel B, lanes 6 and 7). The ligation yield was highest at 12.5–22.5% PEG 6000 condition. Eq. 2 was used for the % yield calculation (see Methods). The production yield of each lane in both panels were indicated with error (standard deviation) shown. nt = nucleotides; n ≥ 3. The full-length gels are presented in Supplementary Fig. 5.

First, we examined the ratio of cDNA:adapter as we anticipated relative concentration of the two will influence ligation efficiency of 5′-adapter ligation. Four cDNA:adapter ratios ranging from 1:1 to 1:10 were performed under 0% PEG 6000 concentration (See Methods). Overall, the production yield increased with decreasing cDNA:adapter ratio, with 6 ± 0% for 1:1 ratio and 42 ± 5% for 1:10 ratio (Fig. 6A).

Next, we examined the effect of PEG on the 5′-adapter ligation efficiency, as we expected PEG will also increase ligation efficiency of 5′-adapter ligation, similar to the 3′-adapter ligation as discussed above (Fig. 1, step 3). As described above, the 5′-adapter ligation efficiency was the highest in 1:10 cDNA:adapter ratio under 0% PEG condition. Hence, 1:10 cDNA:adapter ratio was adapted during the examination. Different concentrations of PEG were investigated, ranged from 0–27.5% (Fig. 6B). Generally, the percentage of yield increased with increasing PEG 6000 concentration as compared to no PEG, and reached to highest at 96 ± 2% under 17.5% PEG 6000 (Fig. 6B). This phenomenon is similar to the 3′-adapter ligation shown above (Fig. 3), suggesting this might be general for ligation reaction. For 5′-adapter ligation, we observed that the yield was >90% under PEG concentration ranged from 12.5% to 22.5%.

Finally, we examined the effect of PEG types on the ligation efficiency of 5′-adapter ligation under the same conditions as described above (Supplementary Fig. 7). Same with that of 3′-adapter ligation reaction as described above (Fig. 1, step 3), PEG 4000, PEG 6000 and PEG 8000 were used. While three types of PEG yielded >90%, they did not show any significant difference (Supplementary Fig. 8, lanes 1–3), indicating that they can be used interchangeably in both 5′ and 3′-adapter ligation reaction.

Overall, we found that the 5′-adapter ligation was most efficient using 1:10 cDNA:adapter ratio and 12.5% to 22.5% PEG concentration at 37 °C for 2 hours.

### Application of improved method on fragmented cellular RNA

To evaluate the efficacy of the optimizations, we produced two new HeLa rG4-seq libraries (HeLa-new-250ng and HeLa-new-50ng) using the new protocol for side-by-side comparison with the HeLa rG4-seq library generated in our previous study^9^. Compared to our previous attempt (HeLa-2016-500ng), the new libraries were constructed using reduced RNA input of 50% (HeLa-new-250ng) and 10% (HeLa-new-50ng) respectively.

Adapter dimers were first quantitated and removed from sequencing data. It was found that the proportion of dimers remain insignificant (between 1.6 to 6.6%) at different levels of input RNA, suggesting a satisfactory performance was given by the optimized adapter removal procedure (Supplementary Table 2). Trimmed reads were successively aligned to hg19 reference genome at mapping rates of 85.3–91.5% (Supplementary Table 3). Since N6 unique molecular identifier (UMI) was newly introduced on the 5′ end of the 3′-adapter sequences, we can evaluate the amount of duplicate reads with this method (Fig. 1). Read duplication rate in HeLa-new-250ng libraries were mild at 18.0–37.0%, suggesting the optimized protocol would enable using half as much input RNA for rG4-seq. Meanwhile, consistent with the high number of PCR amplification cycles required in library preparation, read duplication rate of HeLa-new-50ng libraries (19 cycles) were higher at 59.0–85.6%, compared to HeLa-new-250ng libraries (12 cycles) (Supplementary Table 3). Altogether, our N6 UMI can identify duplicate reads upon increasing PCR cycles and is invaluable in occasions where the amount of input RNA is limited.

**Figure 7:**
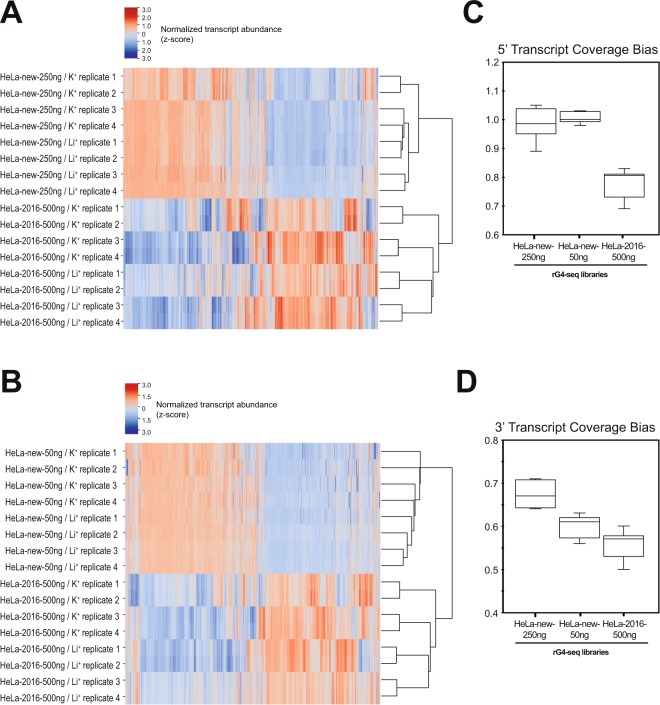
Application of improved method on fragmented cellular RNA. (**A**,**B**) Heatmap and hierarchical clustering results comparing transcript abundances quantitated by sequencing reads in three rG4-seq libraries (HeLa-new-50ng, HeLa-new-250ng, HeLa-2016-500ng). Transcript abundances were measured in TPM (Transcripts per million) and further normalized by z-score across samples. Only mRNA of log_2_(TPM + 1) ≥ 2 were included in the analysis. Results suggested rG4-seq libraries (HeLa-new-250ng, HeLa-50ng) generated with optimized protocol have lower transcript abundance variation between biological replicates when compared to prior arts (HeLa-2016-500ng library) (**C**,**D**) 5′ and 3′ transcript coverage bias comparison between three rG4-seq libraries. Results suggested rG4-seq libraries (HeLa-new-250ng, HeLa-50ng) generated with optimized protocol have lower coverage bias in both 5′ and 3′ transcript ends.

Importantly, new libraries were found to have lower transcript abundance variation between biological replicates. By conducting hierarchical clustering of rG4-seq libraries based on normalized abundance, it was found that HeLa-2016-500ng libraries were preferentially clustered by corresponding biological replicate number rather than the experimental condition (K^+^ or Li^+^) (Fig. 7A,B), suggesting there might be alternative sources of transcript abundance variances apart from ion concentrations during reverse transcription. In contrast, regardless of read duplication rate the new rG4-seq libraries had lower sample variances and were not clearly segregated by replicate number in hierarchical clustering (Fig. 7A,B), indicating the protocol optimizations have mitigated the alternative sources of variances and thus improved sample reproducibility. Notably, it was also revealed that sequencing data of new rG4-seq libraries had lower 5′ and 3′ transcript coverage bias as compared to prior arts (Fig. 7C,D), which allow a better coverage of the entire transcript.

In whole, our findings supported that the proposed optimizations are applicable to sequencing library preparation using fragmented cellular RNA and are associated with improvements in sequencing data quality and consistency. Moreover, the optimized workflow could also be applied to lower RNA input levels by incorporating UMI barcode during library preparation, thus enabling rG4-seq application in scenarios where input RNA material is scarce.

## Conclusion

In this work, we have reported the optimization of 5 key experimental steps (Fig. 1) involved in the cDNA library preparation of rG4-seq (Figs 2–6), and have demonstrated that the improved experimental protocol can be performed in about 6 hours, with lower RNA input, lower transcript abundance variation between biological replicates, and lower 5′ and 3′ transcript coverage bias when compared to prior arts (Fig. 7). Our method and findings are applicable to the library preparation for RNA G-quadruplex studies, and can be potentially applied to other biological applications that require cDNA library preparation.

## Supplementary information

Supplementary Information

Supplementary Table 1

## Data Availability

Sequencing datasets generated in this study are available in the NCBI SRA repository under the accession number PRJNA541052.
