# Author Correction: Systematic evaluation and optimization of the experimental steps in RNA G-quadruplex structure sequencing

**DOI:** 10.1038/s41598-020-60961-z

**Published:** 2020-02-26

**Authors:** Pui Yan Yeung, Jieyu Zhao, Eugene Yui-Ching Chow, Xi Mou, HuiQi Hong, Leilei Chen, Ting-Fung Chan, Chun Kit Kwok

**Affiliations:** 1 Department of Chemistry, City University of Hong Kong, Kowloon Tong, Hong Kong, SAR China; 2 School of Life Sciences, and State Key Laboratory of Agrobiotechnology, The Chinese University of Hong Kong, Shatin, Hong Kong, SAR China; 3 Department of Physiology, Yong Loo Lin School of Medicine, National University of Singapore, Singapore, 117549 Singapore; 4 Cancer Science Institute of Singapore, National University of Singapore, Singapore, 117599 Singapore; 5 Department of Anatomy, Yong Loo Lin School of Medicine, National University of Singapore, Singapore, 117594 Singapore

Correction to: *Scientific Reports* 10.1038/s41598-019-44541-4, published online 30 May 2019

In Figure 7, the sample labels for panels A and B are incorrectly ordered. The correct Figure 7 appears below as Figure 1.

**Figure 1:**
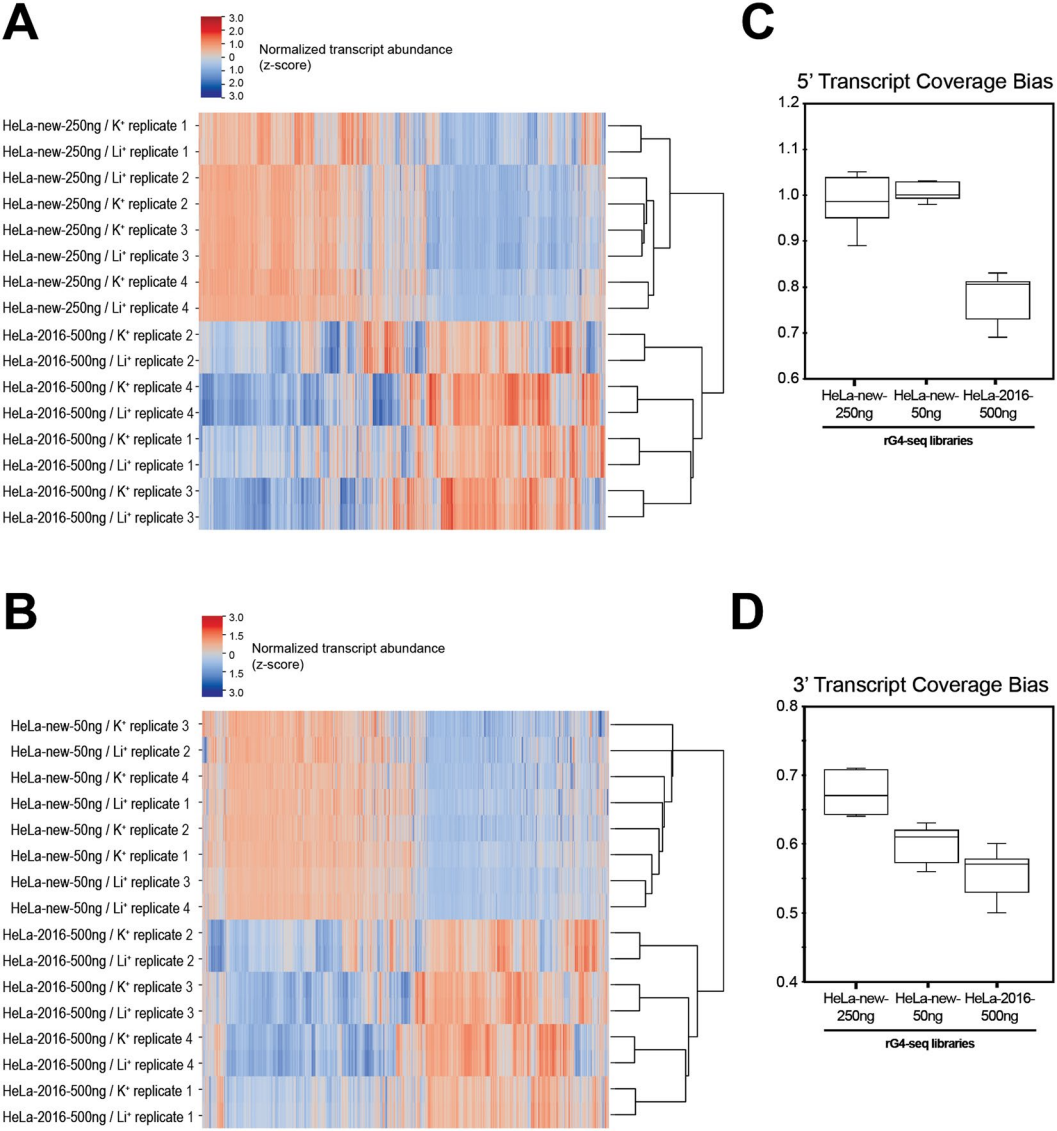
.

