# Supplementary material for: Systematic evaluation and optimization of the experimental steps in RNA G-quadruplex structure sequencing
Source: Sci Rep. 2019 May 30;9:8091. doi: 10.1038/s41598-019-44541-4 (PMC6542847; doi:10.1038/s41598-019-44541-4)
Supplement: Supplementary file 1 — Supplementary Information [file 41598_2019_44541_MOESM1_ESM.pdf]

Systematic evaluation and optimization of the experimental steps in RNA G-quadruplex structure sequencing

Pui Yan Yeung[1,#], Jieyu Zhao[1,#], Eugene Yui-Ching Chow[2,#] , Xi Mou[1], HuiQi Hong[3,4], Leilei Chen[4, 5], Ting-Fung Chan[2,*], Chun Kit Kwok[1, *]

[1] Department of Chemistry, City University of Hong Kong, Kowloon Tong, Hong Kong SAR, China.

[2] School of Life Sciences, and State Key Laboratory of Agrobiotechnology, The Chinese University of Hong Kong, Shatin, Hong Kong SAR, China.

[3] Department of Physiology, Yong Loo Lin School of Medicine, National University of Singapore, Singapore 117549, Singapore.

[4] Cancer Science Institute of Singapore, National University of Singapore, Singapore 117599, Singapore.

[5] Department of Anatomy, Yong Loo Lin School of Medicine, National University of Singapore, Singapore 117594, Singapore.

[#]These authors contributed equally to this work.

[*]Corresponding authors: ckkwok42@cityu.edu.hk (C.K.K) or tf.chan@cuhk.edu.hk (T.F.C)

Supplementary Figure 1. The efficiency of different enzymes on dephosphorylation.

Supplementary Figure 2. The effect of 3' adapter concentration and different percentages of PEG 8000 on the 3' adapter ligation efficiency.

Supplementary Figure 3. The effect of 3' adapter concentration on the 3' adapter ligation efficiency in 17.5% PEG 8000.

Supplementary Figure 4. The effect of different types of PEG on the 3' adapter ligation efficiency.

Supplementary Figure 5. The effect of enzyme digestion and column purification on excess 3' adapters.

Supplementary Figure 6. The effect of different monovalent ion-containing buffers and enzymes on the reverse transcription for pre-miRNA 149 wild-type.

Supplementary Figure 7. The effect of 5' adapter concentration and different PEG 6000 concentrations on the 5' adapter ligation efficiency.

Supplementary Figure 8. The effect of different types of PEG on the 5' adapter ligation efficiency.

Supplementary Table 1. DNA and RNA oligonucleotides used in this study. (attached separately in an excel format)

Supplementary Table 2. Sequencing data statistics of new rG4-seq libraries (HeLa-new-250ng, HeLa-new-50ng).

Supplementary Table 3. Read alignment and duplication statistics.

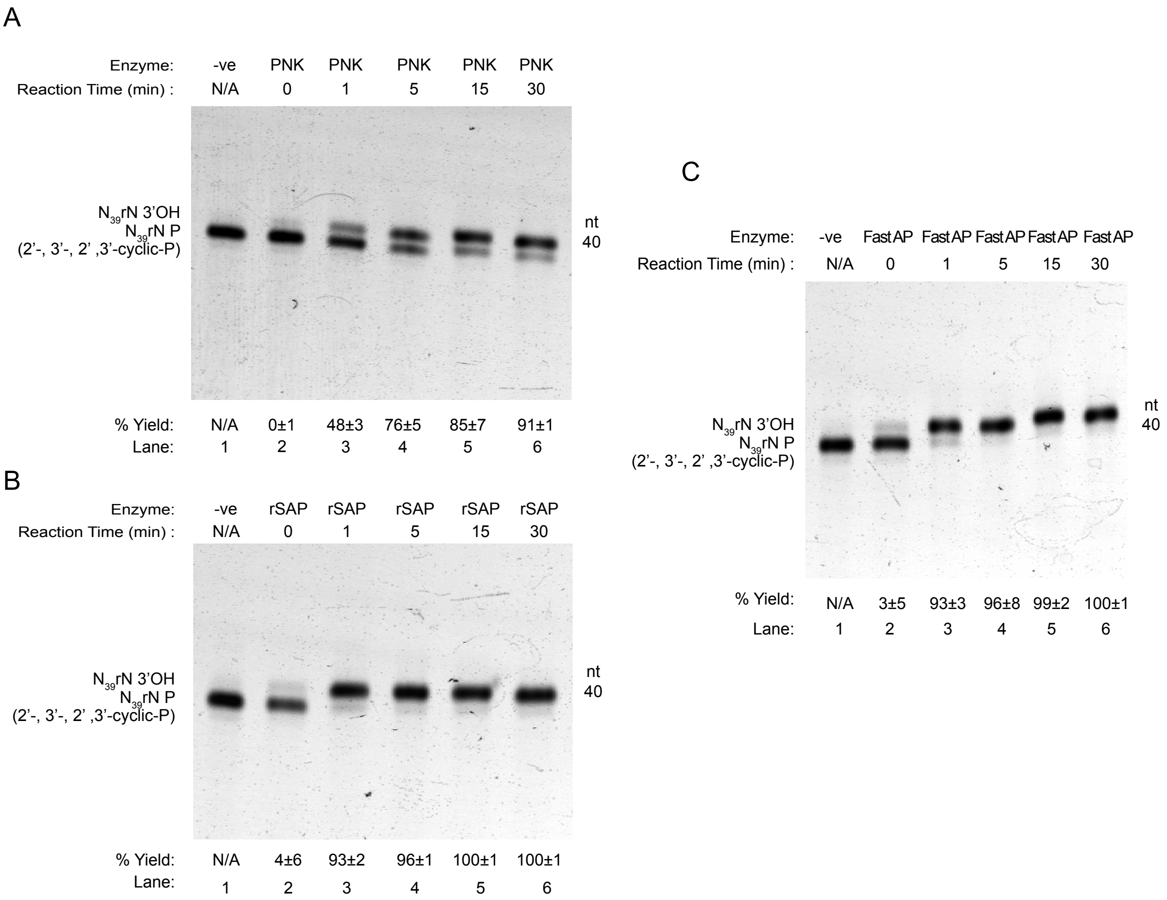

Supplementary Figure 1. The efficiency of different enzymes on dephosphorylation. 40-nt N_{39}rN 3'-P oligonucleotide (with 5' OH and 2',3'-cyclic-P,2'P, 3'P ends) was performed by three enzymes, (A) T4 PNK, (B) rSAP and (C) Fast AP. The reactions were performed in 5 different time-points. The dephosphorylation efficiency was enhanced through longer reaction time (panel A-C, lanes 1-6). Furthermore, as the data suggested, PNK showed a slower efficiency than the other two enzymes. Equation 1 was used for the % yield calculation (See Methods). Errors shown were standard deviation. nt=nucleotide; n=3.

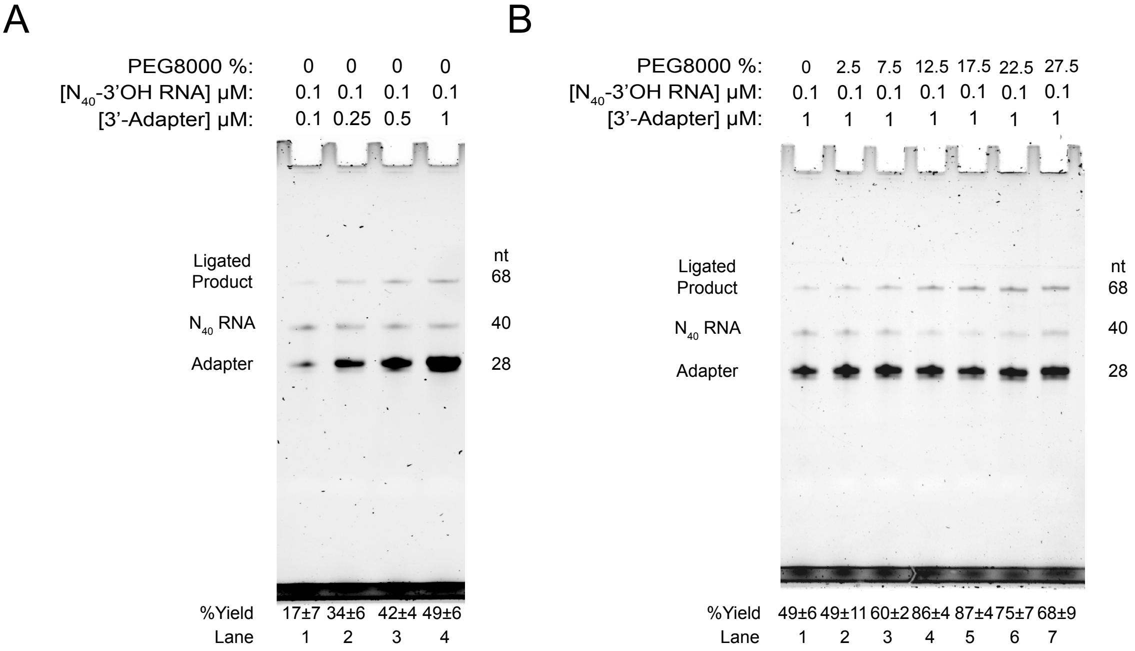

Supplementary Figure 2. The effect of 3' adapter concentration and different percentages of PEG 8000 on the 3' adapter ligation efficiency. The figure showed the efficiency of 3' adapter ligation performed by T4 RNA Ligase 2, truncated KQ with varying (A) RNA:adapter ratio, from 1:1 to 1:10, or (B) the concentrations of PEG 8000, from 0% to 27.5%. The 40-nt RNA N_{40}-3'-OH (with 5'OH and 3'OH ends) was ligated with the 28-nt 3' adapter (with 5'rApp and 3'C3 spacer ends) to produce the 68-nt ligation product. The ligation yield enhanced with increasing 3' adapter concentration (panel A, lane 1-4). From 0% to 17.5% PEG 8000 concentration, the ligation yield increased with increasing PEG 8000 concentration (panel B, lanes 1-5), but started to drop afterwards (panel B, lanes 6 and 7). The ligation yield was the highest at 12.5-17.5% PEG 8000 condition in 1:10 RNA:adapter ratio. Equation 2 was used for the % yield calculation (See Methods). Errors shown were standard deviation. nt=nucleotide; n ≥3.

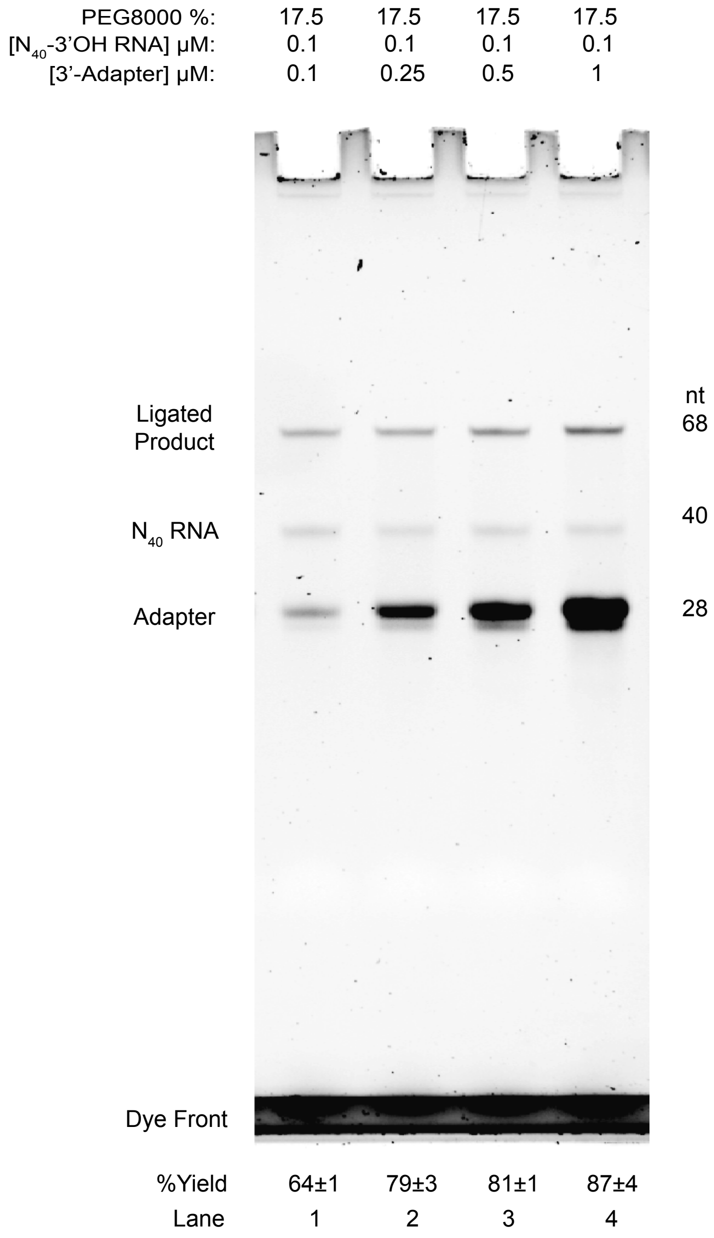

Supplementary Figure 3. The effect of 3' adapter concentration on the 3' adapter ligation efficiency in 17.5% PEG 8000. The above panels showed the efficiency of 3' RNA ligation performed by T4 RNA Ligase 2, truncated KQ with 17.5% of PEG 8000. The 40-nt N_{40}-3'-OH (with 5'OH and 3'OH ends) was ligated with the 28-nt 3' adapter (with 5'rApp and 3'C3 spacer ends) in four RNA:adapter ratios, 1:1, 1:2.5, 1:5 and 1:10, as shown in lanes 1-4 respectively. The size of the ligated product is 68-nt. The ligation yield enhanced with increasing 3' adapter concentration in 17.5% PEG condition. Equation 2 was used for the % yield calculation (See Methods). Errors shown were standard deviation. nt=nucleotide; n ≥3.

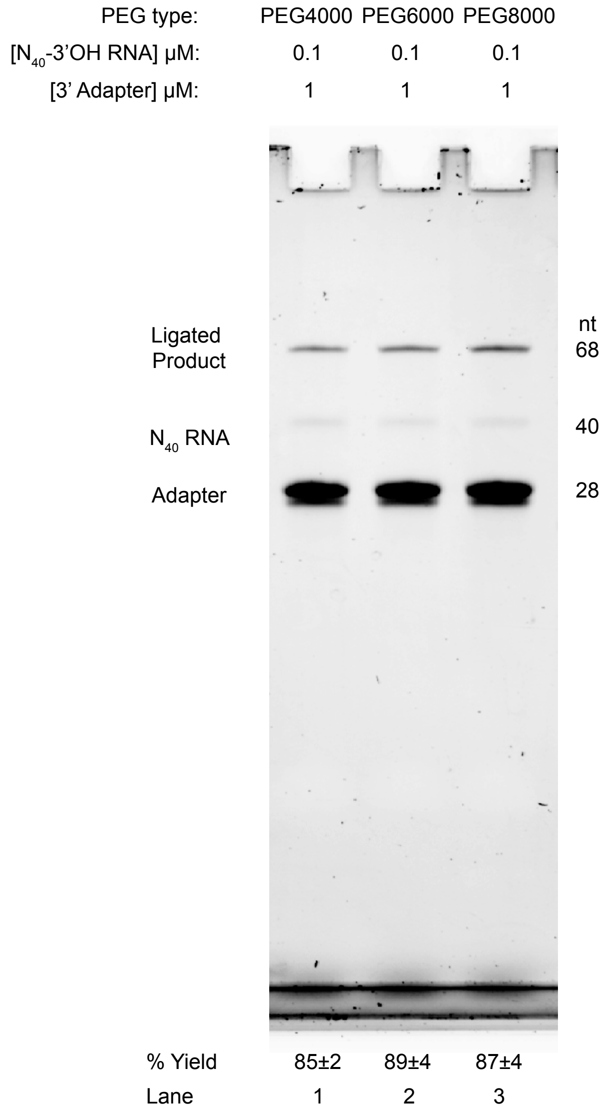

Supplementary Figure 4. The effect of different types of PEG on the 3' adapter ligation efficiency. The figure showed the efficiency of 3' adapter ligation performed by T4 RNA Ligase 2, truncated KQ with three types of PEG: PEG 4000, PEG 6000 and PEG 8000 (lanes 1-3), in 1:10 RNA:adapter ratio and 17.5% PEG condition. As the data suggested, the % yield generated by the three types of PEG were similar and within error. Equation 2 was used for the % yield calculation (See Methods). Errors shown were standard deviation. nt=nucleotide; n ≥3.

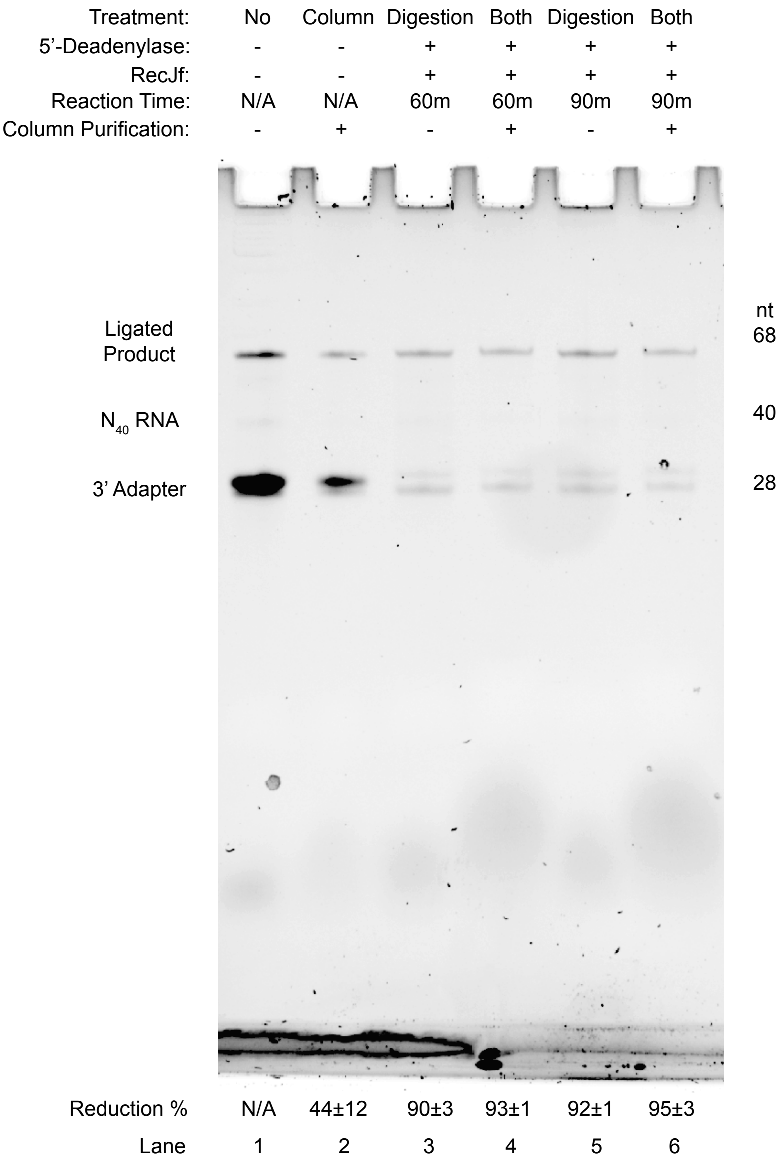

Supplementary Figure 5. The effect of enzyme digestion and column purification on excess 3' adapters. The efficiency of the three approaches to remove the excess 3' adapter (lane 1), column purification only (lane 2), enzymatic digestion for 60 minutes and 90 minutes (lanes 3 and 5), and column purification after enzymatic digestion (lane 4 and 6) were shown. After ligation of N_{40} RNA (40 nt) with 3' adapter (28 nt) to form the ligated product (68 nt), unused adapters remain and need to be removed for subsequent reactions. As compared with no treatment sample in lane 1, column purification alone was able to remove over 40% of excess adapters, while adopting the digestion (lanes 3 and 5) or both digestion and column purification (lanes 4 and 6) removed more than 90% of excess adapters. Furthermore, if the reaction time increased from 60 minutes to 90 minutes, the reduction % enhanced slightly (lanes 3 and 5). Equation 3 was used for reduction % calculation (see Methods). Note that the reduction was calculated by ratio of ratio (see Methods, equation 3), thus the difference observed in the gel image was unaffected by the loading error. The reduction percentage of each treatment was shown with the standard deviations indicated. nt=nucleotide; n=3.

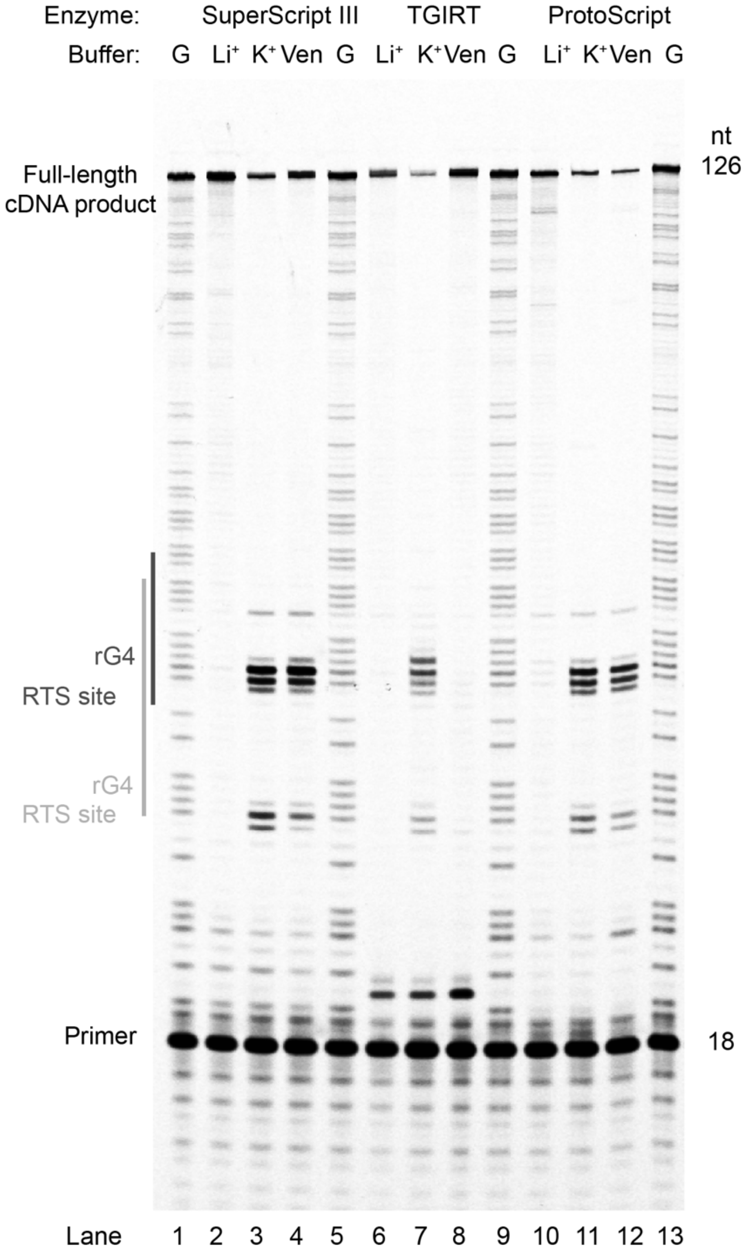

Supplementary Figure 6. The effect of different monovalent ion-containing buffers and enzymes on the reverse transcription for pre-miRNA 149 wild-type. The reverse transcription was conducted with Li^+-, K^+-containing and vendor-provided/recommended buffers from SuperScript III (SSIII) (lanes 2-4), TGIRT (lanes 6-8) and ProtoScript (lanes 10-12). Dideoxycytidine sequencing was performed for guanosine (G) assignment. (lanes 1, 5, 9, 13). The full-length cDNA product band is at 126nt. The primer is at 18 nt. Reverse transcriptase stalling induced by RNA G-quadruplex formation was observed in both K^+ and vendor buffer for SSIII and ProtoScript, yet, only K+ buffer contributed to the reverse transcriptase stalling for TGIRT.

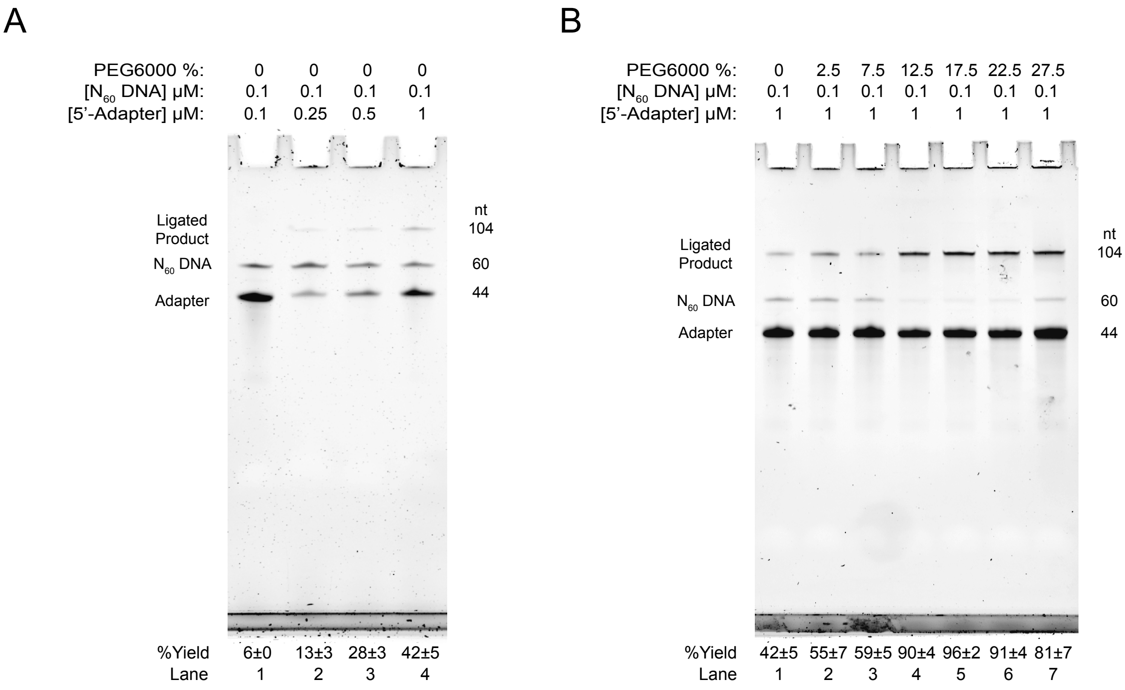

Supplementary Figure 7. The effect of 5' adapter concentration and different PEG 6000 concentrations on the 5' adapter ligation efficiency. The 104-nt ligated products were produced by T4 DNA ligase which either varies in (A) cDNA: adapter ratio, from 1:1 to 1:10, or (B) PEG 6000 percentages, ranged from 0% to 27.5%. The 60-nt N_{60}-DNA (with 5'OH and 3'OH ends) was ligated with 44-nt 5' adapter (with 5'P and 3'C3 spacer ends) to produce 104-nt ligation products. The ligation yield increased with increasing 5' adapter concentration (panel A, lanes 1-4). Based on the results from 0% to 17.5%, the ligation efficiency increased with increasing PEG concentration (panel B, lane 1-5), but started to drop afterwards (panel B, lanes 6 and 7). The ligation yield was highest at 12.5-22.5% PEG 6000 condition. Equation 2 was used for the % yield calculation (see Methods). The production yields of each lane in both panels were indicated with error (standard deviation) shown. nt=nucleotides; n≥3.

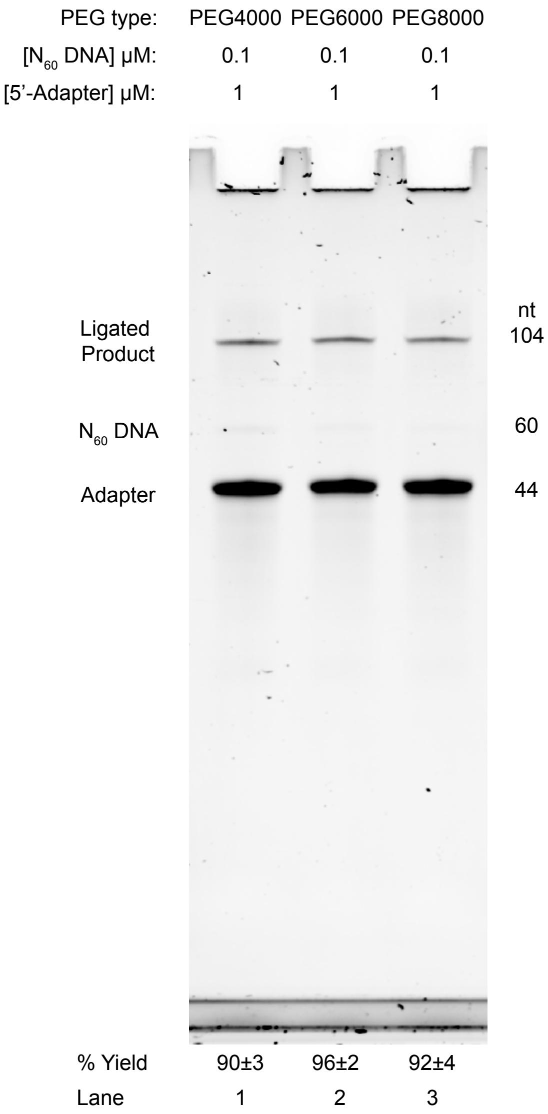

Supplementary Figure 8. The effect of different types of PEG on the 5' adapter ligation efficiency. The figure showed the efficiency of 5' adapter ligation performed by T4 DNA ligase with three types of PEG: PEG 4000, PEG 6000 and PEG 8000 (lanes 1-3), in 1:10 cDNA:adapter ratio and 17.5% PEG condition. As the data suggested, the % yield generated by the three types of PEG were similar and within error. Equation 2 was used for the % yield calculation (See Methods). Errors shown were standard deviation. nt=nucleotide; n≥3

Supplementary Table 1. DNA and RNA oligonucleotides used in this study. (attached separately in an excel format)

Supplementary Table 2. Sequencing data statistics of new rG4-seq libraries (HeLa-new-250ng, HeLa-new-50ng).

**Library**/Sample	**Total sequenced read pairs (M)**	**Adapter dimer read pairs (M)**	**Adapter dimer rate (%)**	**Short (<20bp) read pairs (M)**	**Short read pair rate (%)**
**HeLa-new-250ng /** K^+ replicate 1	165.9	4.5	2.7%	1.3	0.8%
**HeLa-new-250ng /** K^+ replicate 2	133.2	8.8	6.6%	2.2	1.6%
**HeLa-new-250ng /** K^+ replicate 3	126.3	6.3	5.0%	4.7	3.7%
**HeLa-new-250ng /** K^+ replicate 4	118.8	1.9	1.6%	6.5	5.5%
**HeLa-new-250ng /** Li^+ replicate 1	118.5	5.8	4.9%	1.7	1.4%
**HeLa-new-250ng /** Li^+ replicate 2	74.2	4.4	5.9%	1.9	2.6%
**HeLa-new-250ng /** Li^+ replicate 3	140.5	3.9	2.8%	5.1	3.6%
**HeLa-new-250ng /** Li^+ replicate 4	111.0	1.9	1.7%	2.9	2.7%
**HeLa-new-50ng /** K^+ replicate 1	85.9	4.1	4.8%	0.8	0.9%
**HeLa-new-50ng /** K^+ replicate 2	167.6	5.1	3.0%	0.6	0.3%
**HeLa-new-50ng /** K^+ replicate 3	130.3	4.7	3.6%	2.1	1.6%
**HeLa-new-50ng /** K^+ replicate 4	75.4	2.9	3.8%	0.6	0.8%
**HeLa-new-50ng /** Li^+ replicate 1	108.9	3.2	3.0%	0.4	0.4%
**HeLa-new-50ng /** Li^+ replicate 2	117.8	2.8	2.4%	0.7	0.6%
**HeLa-new-50ng /** Li^+ replicate 3	88.6	3.1	3.5%	0.8	0.9%
**HeLa-new-50ng /** Li^+ replicate 4	187.4	5.5	2.9%	1.4	0.8%

Adapter dimer read pairs were defined as read pairs with 1 or 0 bases remaining after adapter trimming, while short read pairs were defined as those with 2-19 bases remaining.

Supplementary Table 3. Read alignment and duplication statistics.

**Library**/Sample	**Total usable read pairs (M)**	**Mapped read pairs (M)**	**Mapping rate (%)**	**Read duplication rate (%)**
**HeLa-new-250ng** / K^+ replicate 1	160.1	137.5	85.9%	18.0%
**HeLa-new-250ng** / K^+ replicate 2	122.2	106.6	87.2%	37.0%
**HeLa-new-250ng** / K^+ replicate 3	115.3	100.6	87.3%	35.0%
**HeLa-new-250ng** / K^+ replicate 4	110.4	98.7	89.4%	21.1%
**HeLa-new-250ng** / Li^+ replicate 1	111.1	93.5	84.1%	26.1%
**HeLa-new-250ng** / Li^+ replicate 2	67.9	59.2	87.2%	29.4%
**HeLa-new-250ng** / Li^+ replicate 3	131.5	115.7	88.0%	33.4%
**HeLa-new-250ng** / Li^+ replicate 4	106.1	97.1	91.5%	20.3%
**HeLa-new-50ng** / K^+ replicate 1	81.0	70.6	87.1%	59.0%
**HeLa-new-50ng** / K^+ replicate 2	162.0	139.4	86.1%	85.6%
**HeLa-new-50ng** / K^+ replicate 3	123.5	105.9	85.8%	83.5%
**HeLa-new-50ng** / K^+ replicate 4	71.9	61.3	85.3%	77.9%
**HeLa-new-50ng** / Li^+ replicate 1	105.3	91.1	86.6%	82.7%
**HeLa-new-50ng** / Li^+ replicate 2	114.3	99.4	87.0%	85.3%
**HeLa-new-50ng** / Li^+ replicate 3	84.7	73.9	87.2%	56.1%
**HeLa-new-50ng** / Li^+ replicate 4	180.5	157.7	87.4%	75.4%

Read pairs with ≥20 bases remaining after adapter trimming are considered usable. Read duplication rate were evaluated using only uniquely-mapped read pairs.
